# Hodgkin Lymphoma Presenting as Pel–Ebstein Fever: A Case Report

**DOI:** 10.1002/cnr2.70039

**Published:** 2024-11-20

**Authors:** Ujjawal Kumar Shriwastav, Deepak Sundriyal, Parmod Kumar, Amit Sehrawat

**Affiliations:** ^1^ Chitwan Medical College Bharatpur India; ^2^ Department of Internal Medicine All India Institute of Medical Sciences Rishikesh India; ^3^ Department of Medical Oncology, Hematology All India Institute of Medical Sciences Rishikesh India

**Keywords:** Hodgkin lymphoma, malignancy‐related fever, Pel–Ebstein fever

## Abstract

**Background:**

Prolonged fever of more than a year duration can be a symptom of underlying malignancy. This article appreciates the need for diligent history taking and carefully performed clinical examination, which is fundamental to reaching an initial diagnosis.

**Case:**

We report the case of a middle‐aged male who presented to us with a fever of more than a year duration. The patient's residence was located in a remote area where the nearby tertiary care center was almost 250 km away. He was empirically treated with antibiotics without any success by local physicians. After more than a year, he reported to us and was diagnosed with Hodgkin lymphoma with a characteristic Pel–Ebstein pattern of fever missed earlier by the local physicians. His fever resolved within 5 days of chemotherapy, and he achieved complete remission.

**Conclusion:**

Meticulous history‐taking and clinical examination are essential for reaching a clinical diagnosis. Malignant causes should be considered in the differentials of prolonged fever.

## 1 Introduction

A fever of more than a year is seldom encountered in clinical practice. It is because of the availability of improved diagnostic modalities. However, one can come across a similar situation in areas where access to a tertiary care center is not readily available. We report a patient with a fever of more than a year duration. A meticulous history is essential for the diagnosis while evaluating a case of fever. Differential diagnosis of underlying malignancy should be considered while encountering a typical relapsing–remitting pattern of Pel–Ebstein fever with a prolonged history, as the delay in diagnosis can lead to poorer outcome and prognosis [1, 2]. This case is unique as it signifies the importance of a good history taking and clinical examination in reaching to a diagnosis at the earliest thus improving the clinical outcomes.

## 2 Case Presentation

A 44‐year‐old male residing in a remote hilly area of Uttarakhand presented to All India Institute of Medical Sciences Rishikesh, India in March, 2020 with a high‐grade fever of more than a year duration. He was a factory worker and was treated empirically with antibiotics with suspicion of enteric fever on multiple occasions (seven times) by local physicians before coming to us. The nearest tertiary care center to his home was 250 km away, so it was difficult for him to visit the higher center. He revealed that he had lost almost 15 kg of weight in the last 6 months on further interrogation. He also noticed multiple swellings on both sides of the neck in the last 6 months. The fever would rise for 5–7 days, followed by an afebrile period of similar duration, suggesting a typical Pel–Ebstein pattern. On examination, bilateral cervical and supraclavicular lymph nodes were enlarged, with a soft consistency. Systemic examination was unremarkable except for moderate splenomegaly.

**FIGURE 1:**
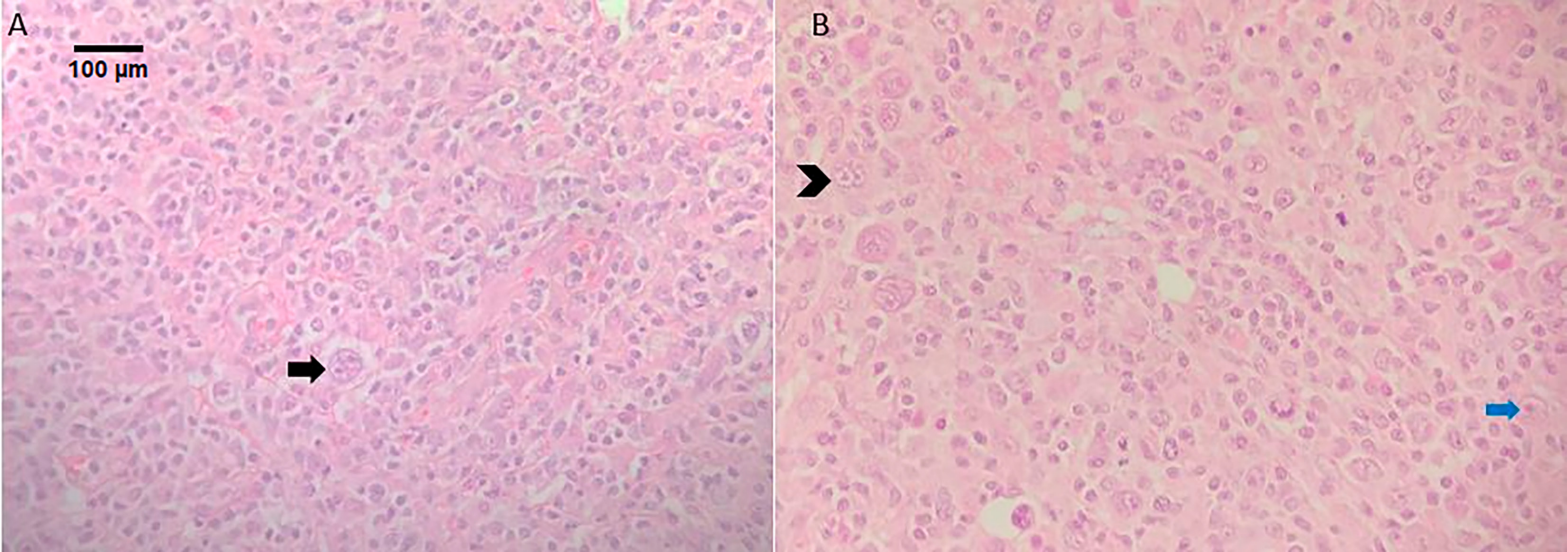
Hematoxylin and Eosin stained sections show effacement of lymph node architecture by lymphoma cells. Numerous bi‐nucleate (arrow—A), mono‐nucleate (blue arrow—B), and multi‐nucleate (arrowhead—B) Reed‐Sternberg cells are seen (400×).

His blood counts revealed anemia and thrombocytopenia [hemoglobin 9.0 (range 12–16) gm/dL, platelets 1.2 (range 1.5–4.5) lac/cumm]. Serum albumin was low, 3 g/dL (range 3.5–5.5 g/dL), serum alkaline phosphatase (ALP), and serum lactate dehydrogenase (LDH) were elevated, 910.7 (range 40–120) U/L, and 907.7(range 120–250) U/L, respectively. An excision lymph node biopsy was done; histopathological examination revealed a polymorphous population of cells with numerous bi‐nucleate, mono‐nucleate, and multi‐nucleate Reed‐Sternberg cells interspersed in between (Figure 1). In immunohistochemistry, the Reed‐Sternberg cells were immuno‐positive for CD15, CD30, and EBV‐LMP1 favoring a diagnosis of Hodgkin lymphoma, mixed cellularity type.

A positron emission tomography‐computed tomography (PET‐CT) scan suggested metabolically active lymph nodes on both sides of the diaphragm with splenic and marrow involvement and without any bulky site. His pulmonary function test and echocardiogram were normal. Tumor lysis syndrome metabolic panel was normal.

We made a final diagnosis of Hodgkin lymphoma, mixed cellularity type, Stage IV, International prognostic score (IPS) 4.

**FIGURE 2:**
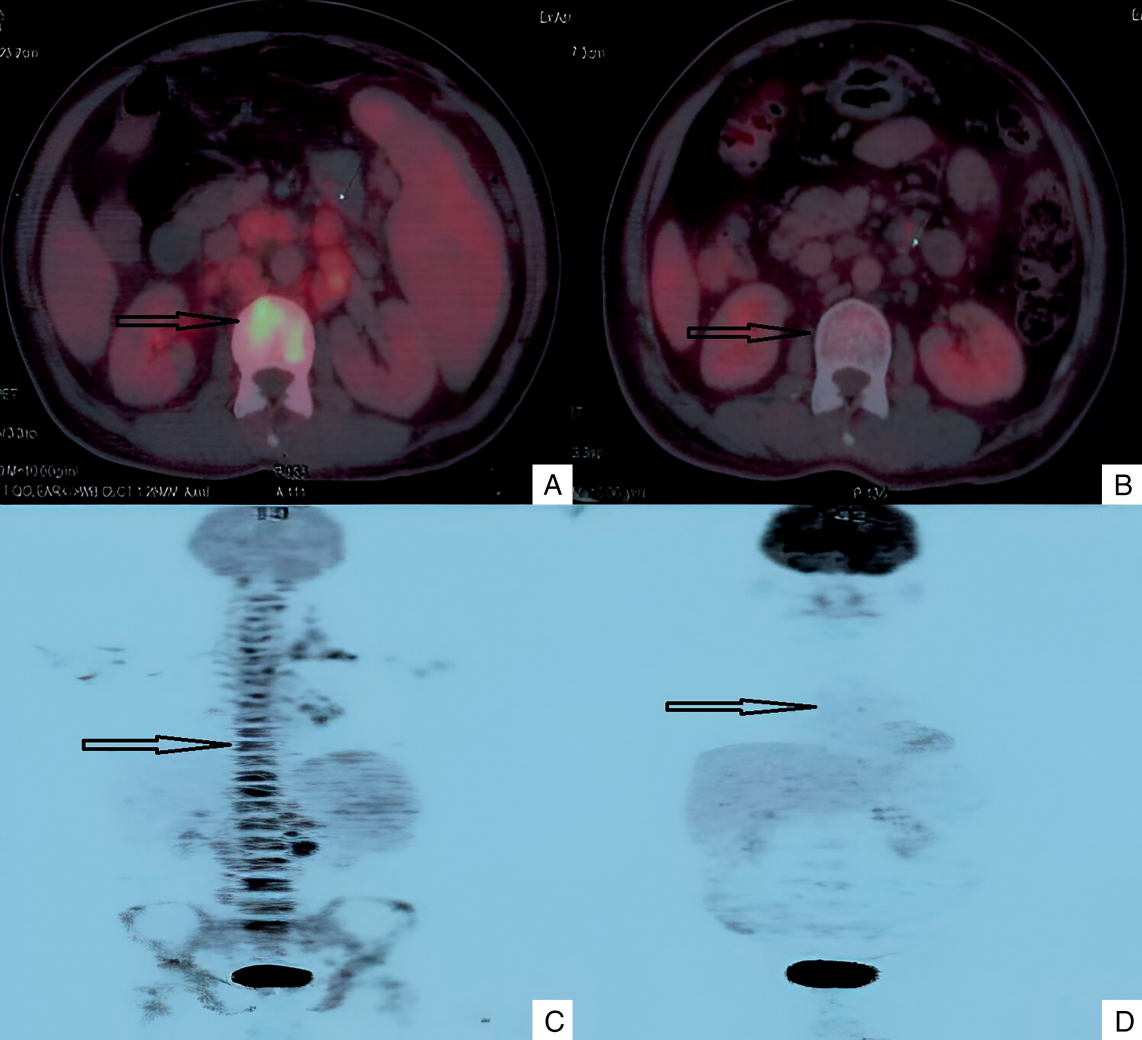
Pre (A) and post (B) treatment PET, and corresponding pre (C) and post (D) treatment CT images suggesting a significant response (arrow) to the therapy.

We planned ABVD protocol therapy. He received pneumococcal and anti‐influenza vaccination 2 weeks prior commencing chemotherapy. He also received oral naproxen, 250 mg not more than twice daily for the control of fever, during this period. Prophylactic allopurinol was given. He received 2 cycles of ABVD (adriamycin 25 mg/m^2^, bleomycin 10 mg/m^2^, vinblastine 6 mg/m^2^, dacarbazine 375 mg/m^2^ on Days 1 and 15 of a 28‐day cycle) protocol followed by an interim evaluation by PET‐CT. PET‐CT was suggestive of a Deauville score of 1 at all sites of disease except a score of 3 at a solitary mediastinal lymph node suggesting a significant response to therapy (Figure 2). Blood counts revealed a rise of hemoglobin to 11.2 g/dL and normalization of serum ALP and serum LDH values. In view of good response, treatment was de‐escalated to AVD (adriamycin, vinblastine, dacarbazine) protocol [3]. He received 4 cycles of AVD protocol. PET‐CT evaluation after the completion of treatment was suggestive of a complete metabolic response. He was advised to follow up at 3 monthly intervals. A clinical examination was performed every 3 monthly interval and PET‐CT was performed once every 6 months. The patient is in clinical and metabolic remission till now.

## 3 Discussion

The distinctive feature in this case was the prolonged history of a cyclical pattern of fever which was missed earlier. A meticulous history taking by the local physicians would have raised suspicion of a fever arising due to malignancy followed by an early referral to a higher center. This would have been proven logistically more appropriate simultaneously reducing the patient's agony. By the time patients reached to us, lymphoma had already spread to the marrow which was evident in the form of cytopenias (low hemoglobin and platelets) and marrow involvement on PET‐CT. It is important to know that a delay in diagnosing a malignancy can lead to the upstaging of the disease, increased morbidity, logistic difficulties, poorer prognosis and outcome. Howell et al. [4, 5] reported that time‐to‐diagnosis of hematological malignancies could be prolonged, reason being multiple general physicians consultations leading to increasing complications at diagnosis. Likewise, Lamb et al. [5] suggested that survival in Hodgkin lymphoma could be improved by around 4% by earlier diagnosis.

In association with transient swelling of lymph nodes and spleen, relapsing–remitting episodes of fever were first noted by Hodgkin in the year 1832 [1]. Pel–Ebstein fever was described separately by Professor Pieter Klaesz Pel and Wilhelm Ebstein in combination with splenomegaly and enlarged lymph nodes [2]. Although the cyclical pattern of Pel–Ebstein fever has been historically described in association with Hodgkin lymphoma, it can also be seen in infectious conditions [6, 7, 8]. Pel–Ebstein fever is seldom encountered in clinical practice; however, the typical presentation of high‐grade fever for a few days followed by an afebrile period and so on should be taken with a pinch of salt, and a religious effort should be made to search for the underlying lymphoma. Other causes of malignancy‐related fever include renal cell carcinoma, myeloproliferative disorders, acute myeloid leukemia, myeloma, hairy cell leukemia, hepatocellular carcinoma, and carcinoma of the ovary. The most likely explanation for the malignancy‐related fever is the release of cytokines from the tumor tissue due to tumor necrosis or the surrounding inflammatory milieu like interkeukin 1 and 6, tumor necrosis factor‐alpha, and interferon [9, 10]. Diagnostic criteria for neoplastic fever as proposed by Zell and Chang [11] have been given in Table 1. It is crucial to rule out non‐neoplastic causes of fever like infections, inflammatory diseases, drug‐related fever, thyroiditis and factitious fever [12]. In a developing nation like India, we seldom encounter undiagnosed fever of prolonged duration. Ineffective primary health care system, shortage of tertiary care centers in rural areas, and socioeconomic factors are some of the reasons for the delay in access to health facility [13, 14]. However, it is gratuitous to say that history‐taking and clinical examination are of paramount importance in reaching a diagnosis.

**TABLE 1:** Diagnostic criteria for neoplastic fever.

1	Temperature over 37.8°C at least once each day
2	Duration of fever over 2 weeks
3	Lack of evidence of infection on A. Physical examination B. Laboratory examinations, e.g., sputum smears or cultures, cultures of blood, urine, stool, bone marrow, spinal fluid, pleural fluid, and discharge from local lesions C. Imaging studies, e.g., chest radiograph and computed tomographic scans of the head, abdomen, and pelvis
4	Absence of allergic mechanisms, e.g., drug allergy, transfusion reaction, and radiation or chemotherapeutic drug reaction
5	Lack of response of fever to an empiric, adequate antibiotic therapy for at least 7 days
6	Prompt, complete lysis of fever by the naproxen test with sustained normal temperature while receiving naproxen

## 4 Conclusion

A meticulous history‐taking and clinical examination is a sine qua non for clinical decision making, which further streamlines the path of accurate diagnosis and early administration of an effective treatment.

A history of prolong fever with cyclical pattern should always be considered in the differential diagnosis of a malignancy related fever.

## Author Contributions

**Ujjawal Kumar Shriwastav:** conceptualization, writing – original draft, writing – review and editing. **Deepak Sundriyal:** conceptualization, methodology, writing – review and editing, formal analysis. **Parmod Kumar:** investigation, writing – original draft, data curation. **Amit Sehrawat:** conceptualization, methodology, writing – review and editing.

## Ethics Statement

IRB approval was not required for this article.

## Consent

We obtained a written informed consent from the patient for the publication of this article.

## Conflicts of Interest

The authors declare no conflicts of interest.

## Data Availability

The data that support the findings of this study are available from the corresponding author upon reasonable request.
